# Advances in 3D-Printed scaffolds for bone defect repair: material strategies and synergistic functional performance

**DOI:** 10.3389/fbioe.2025.1707406

**Published:** 2025-11-12

**Authors:** Xijie Tang, Haijia Xu, Xiangzhong Liu, Yi Yang, Zhanghua Li

**Affiliations:** 1 Department of Orthopaedics, Wuhan Third Hospital, Tongren Hospital of Wuhan University, Wuhan, China; 2 Hubei Key Laboratory of Exercise Training and Monitoring, Department of Sports Medicine, Wuhan Sports University, Wuhan, China

**Keywords:** 3D printing, bone tissue engineering, polymer–ceramic composites, mechanical properties, degradation, clinical translation

## Abstract

Large bone defects remain a major clinical challenge, as traditional grafts and implants often fail to provide both long-term stability and biological integration. Three-dimensional (3D) printing offers unique advantages in fabricating patient-specific scaffolds with controlled architectures, enabling precise modulation of mechanics, degradation, and biological function. Natural and synthetic polymers, ceramics, and their composites have been widely explored, while strategies such as nanofiller reinforcement, surface modification, and growth-factor delivery further enhance osteogenesis, angiogenesis, immunomodulation, and anti-infection performance. This review systematically summarizes recent progress in 3D-printed biomaterial scaffolds for bone defect repair, focusing on their mechanical properties, degradation behavior, bioactivity, infection resistance, and vascularization. Current advances highlight how multifunctional design and material–biological coupling can bridge the gap between laboratory research and translational applications. Future directions emphasize material innovation, hierarchical scaffold design, and clinical standardization to accelerate the safe and effective application of 3D-printed scaffolds in bone regeneration.

## 1 Introduction

**FIGURE 1:**
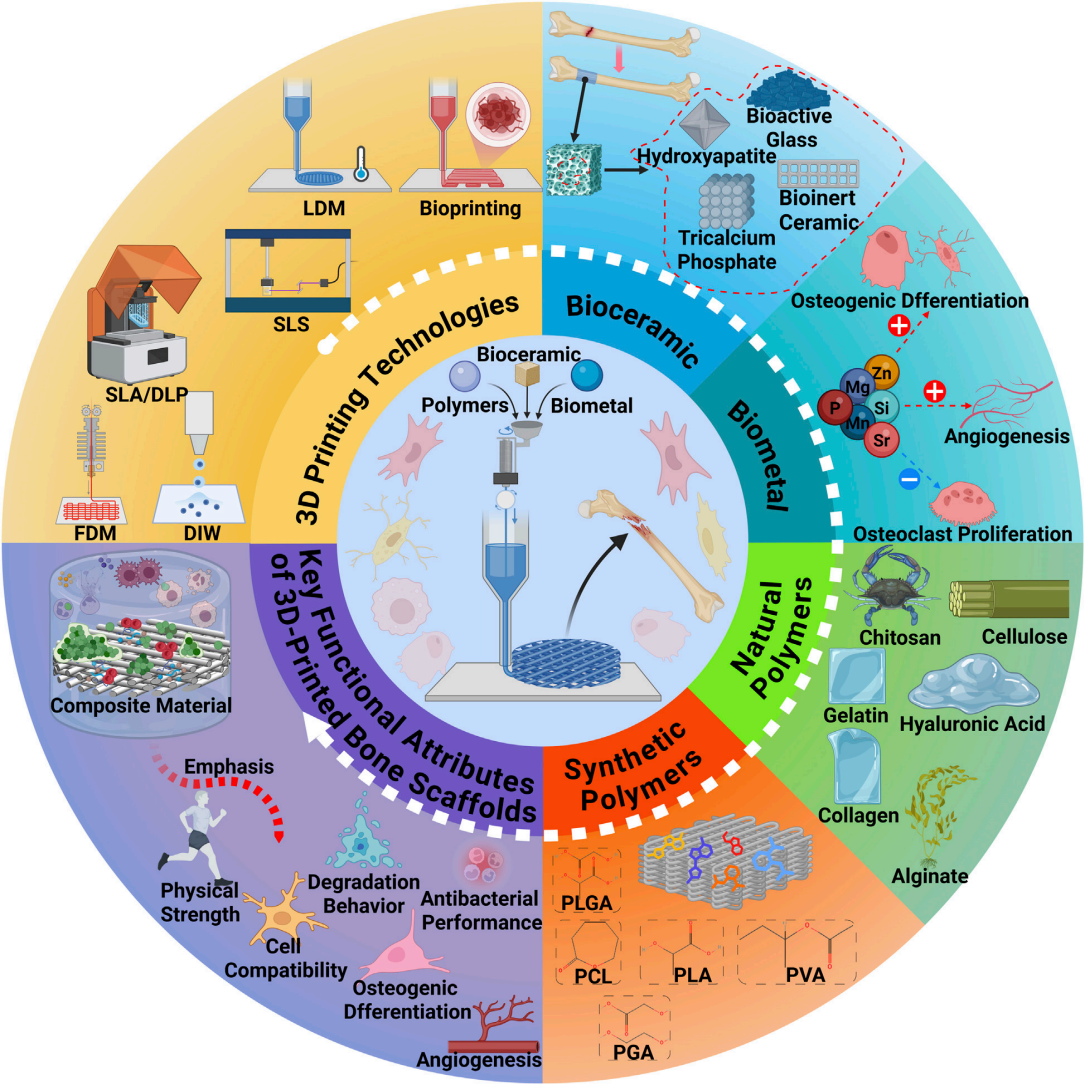
Overview of the 3D Printing Technologies and Polymeric Materials for Bone Tissue Engineering. This figure was drawn using Biorender (https://www.biorender.com/).

Bone defects caused by trauma, tumor resection, infection, or congenital conditions remain a major clinical challenge (Hoveidaei et al., 2025). Critical-sized segmental defects frequently progress to delayed union or nonunion and remain difficult to reconstruct effectively with current interventions, severely compromising patients’ quality of life and functional recovery (Koons et al., 2020). Although autografts and allografts are widely employed, their utility is limited by donor-site morbidity, restricted availability, immunological complications, and inconsistent long-term outcomes (Collon et al., 2021). These limitations underscore the need for materials and strategies that deliver immediate mechanical stability while supporting subsequent vascularized bone regeneration and remodeling (Zhu et al., 2021). An overview of the 3D printing technologies and polymeric materials used in bone tissue engineering is illustrated in Figure 1.

**FIGURE 2:**
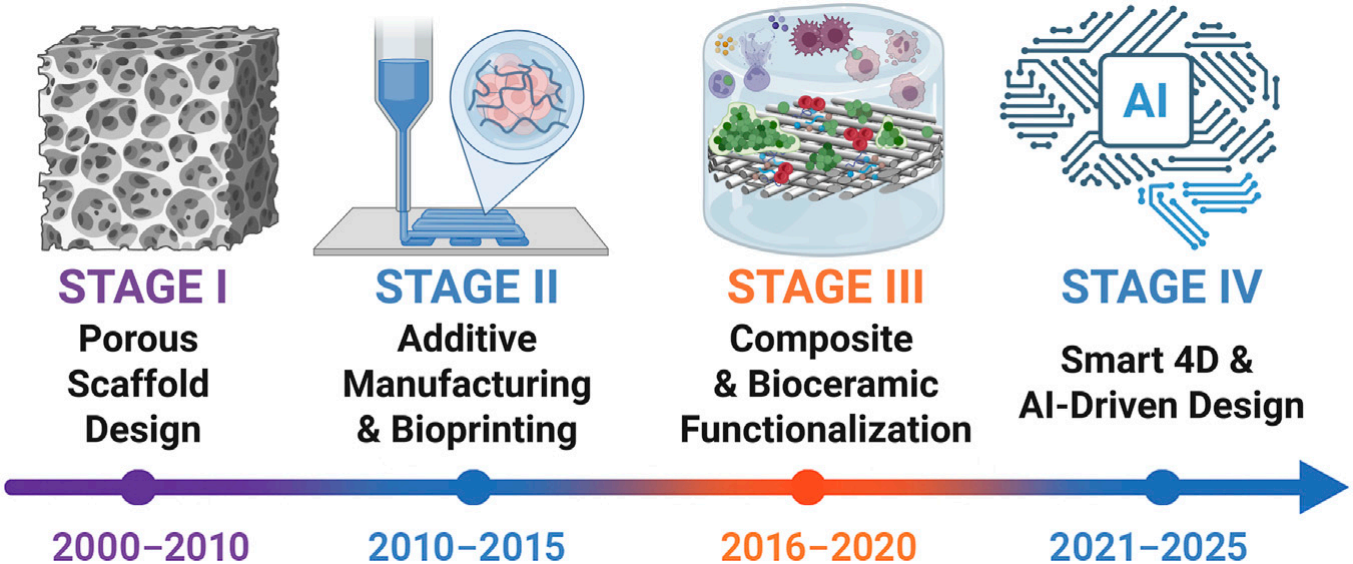
Timeline of 3D printing evolution in bone tissue engineering (2000–2025). Four stages are outlined: porous scaffold design (Hollister, 2005; Zadpoor and Malda, 2017), additive manufacturing and bioprinting (Do et al., 2015; Murphy and Atala, 2014), composite and bioceramic functionalization (Ma et al., 2018; Wang et al., 2020), and the emergence of smart 4D and AI-driven strategies (Wang et al., 2024; Yuan Y. et al., 2024). This figure was drawn using Biorender (https://www.biorender.com/).

Bone tissue engineering (BTE) provides a framework that integrates scaffolds, cells, and biochemical cues to recapitulate key features of the bone microenvironment (Seunghun et al., 2022). Advances in three-dimensional (3D) printing (additive manufacturing) now enable defect-matched, patient-specific architectures with prescribed pore size, interconnectivity, and anisotropy (Qu et al., 2021). By coupling geometry with material selection, structural and transport properties can be engineered with greater precision and reproducibility than conventional methods, thereby improving guidance of cell migration, vascular ingrowth, and load transfer (Giannitelli et al., 2015; Hussey et al., 2018; Picado-Tejero et al., 2025). In translational contexts, architecture should also be matched to a printable processing window and sterilization route to preserve fidelity and function (Del-Mazo-Barbara et al., 2024; Schwab et al., 2020). The evolution of 3D printing in bone tissue engineering from early porous scaffold design to smart 4D and AI-driven strategies is summarized in Figure 2.

The selection of suitable biomaterials is a pivotal aspect of BTE. Materials that mimic the structural and biological characteristics of natural bone must exhibit biocompatibility, bioactivity, biodegradability, and appropriate mechanical properties (Bandyopadhyay et al., 2023). Polymeric materials, in particular, have gained widespread application in bone regeneration due to their tunable physicochemical properties, excellent processability, and favorable biocompatibility (Pourhajrezaei et al., 2024). In parallel, bioactive ceramics and metallic biomaterials are often incorporated—as reinforcing or functional phases within composites—to reconcile osteoconductivity with load-bearing requirements and to better manage degradation and integration.

Each type of polymer has inherent advantages and limitations. Natural polymers offer superior biocompatibility, bioactivity, and biodegradability, yet they often lack mechanical strength and exhibit inconsistent degradation profiles, limiting their ability to provide sustained structural support and controlled release of bioactive cues (Janmohammadi et al., 2023; Kravanja and Finšgar, 2022; Tiwari et al., 2023). Conversely, synthetic polymers possess adjustable mechanical and degradation properties through molecular design and exhibit excellent processability, but they typically fall short in terms of intrinsic bioactivity (Abbasi et al., 2020; Swarupa and Thareja, 2024). To address these limitations, surface modification and composite material strategies have been employed, enabling fine-tuning of material properties and integration of complementary functionalities to meet the complex demands of bone regeneration (Acharjee et al., 2024; Sanjarnia et al., 2024).

In addition to structural design and material selection, controlled delivery of bioactive ions or small molecules has been explored to regulate immune response, angiogenesis, osteogenesis, and antimicrobial activity at different healing stages. Bioactive ions play essential roles in modulating cellular behaviors during bone regeneration, including proliferation, differentiation, and matrix mineralization (Lin et al., 2019; Lin et al., 2021). In recent years, considerable progress has been made in developing ion delivery systems, particularly those based on polymer matrices for controlled ion release, which have shown great promise in enhancing osteogenesis and improving bone quality (Luo et al., 2024; Mao et al., 2024; Wei et al., 2023; Xu et al., 2022; Zhou et al., 2024). Furthermore, ions such as calcium, phosphate, and silicon have demonstrated potent osteoinductive effects, accelerating bone formation and contributing to long-term regenerative outcomes (Zheng et al., 2021). Therefore, engineering polymeric scaffolds with tailored ion release capabilities represents a powerful strategy to enhance both the efficacy and stability of bone defect repair.

Given that systematic evaluations of polymer-based 3D-printed scaffolds for bone defect repair remain insufficient, this review focuses on recent advances in this field. Particular attention is devoted to the interplay among structural design, mechanical performance, degradation kinetics, and microenvironmental regulation, with an emphasis on how these factors act synergistically to achieve bioadaptive bone regeneration. In particular, this review highlights an integrative perspective that considers mechanical reinforcement, controlled degradation, immunomodulation, and angiogenesis in the unified context of 3D printing strategies. Specifically, the review focuses on recent progress in material design strategies for 3D-printed scaffolds and their effects on biological performance in bone regeneration. Furthermore, translational challenges and potential clinical applications are briefly discussed to provide a comprehensive overview from material innovation to clinical implementation, while also outlining future trends such as 4D printing, bio-inks containing living cells, and AI-assisted scaffold design, which underscore the importance of interdisciplinary integration across materials science, biology, and clinical medicine in driving the next-generation of adaptive bone repair systems.

## 2 3D printing technology

Three-dimensional (3D) printing has rapidly developed as a key enabling technology in bone tissue engineering, enabling patient-specific scaffolds with complex geometries and tunable porosity (MacDonald and Wicker, 2016; Wickramasinghe et al., 2020). Compared with traditional fabrication routes, it provides precise control over architecture and internal connectivity to better match biological and mechanical requirements (MacDonald and Wicker, 2016; Prendergast and Burdick, 2020). This capability is particularly relevant for bone repair, where early stability, nutrient transport, and vascularization are all essential (Guzzi and Tibbitt, 2020). Various printing techniques have therefore been developed, each characterized by distinct processing principles, material compatibility, and structural performance (Bandyopadhyay et al., 2020; Du X et al., 2019).

### 2.1 Extrusion-based printing

**FIGURE 3:**
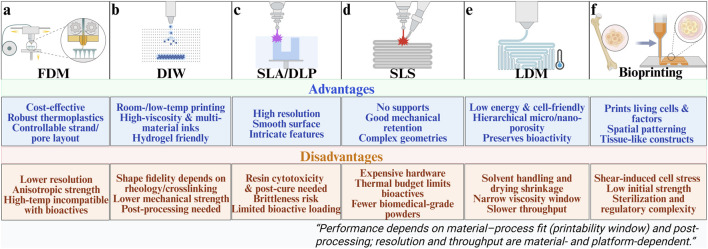
Various 3D printing process schematics and their advantages and disadvantages. **(a)** Fused deposition modeling. **(b)** Direct ink writing. **(c)** Stereolithography/Digital light processing. **(d)** Selective laser sintering. **(e)** Low-temperature Deposition Manufacturing. **(f)** Bioprinting. This figure was drawn using Biorender. (https://www.biorender.com/).

Extrusion-based platforms dispense a melt, slurry, or viscoelastic ink through a nozzle and solidify filaments layer by layer to build lattice architectures (Chen et al., 2020; Tan et al., 2022). They are widely used for bone-regeneration scaffolds because strand spacing and layer height can be directly mapped onto target structures with relatively accessible hardware (Babilotte et al., 2019). The principal modes are fused deposition modeling (FDM) for thermoplastics and direct ink writing (DIW) for viscoelastic pastes or hydrogel inks (Awasthi and Banerjee, 2021). In FDM, nozzle diameter and raster spacing determine strut size and pore anisotropy, whereas layer height and thermal history govern interlayer bonding and crystallinity (Figure 3a) (Winarso et al., 2022; Zhang et al., 2023).

In DIW, print fidelity depends strongly on rheological behavior, requiring shear-thinning flow inside the nozzle together with rapid elastic recovery after deposition (Ma et al., 2021; Saadi et al., 2022) If recovery is insufficient, filaments spread or sag and structural accuracy deteriorates (Ma et al., 2021; Zandi et al., 2021). By contrast, well-balanced viscoelastic properties promote shape retention and enable accurate reproduction of the designed architecture (Figure 3b) (Jamee et al., 2021; Parodi et al., 2023). Common thermoplastics (PLA, PCL, PLGA) and their ceramic-filled blends are frequently employed in bone defect repair (Kirillova et al., 2021). Such filament-controlled microarchitectures promote osteoblast adhesion and accelerate interfacial mineralization, facilitating early osteointegration and stable mechanical load transfer at the defect site (Bin et al., 2025; Turnbull et al., 2018).

### 2.2 Laser-based printing

Stereolithography (SLA) scans a UV/visible beam to polymerize photocurable resins voxel by voxel, while digital light processing (DLP) projects entire layer images for higher throughput (Figures 3c,d) (Daly et al., 2021). These techniques provide high resolution and enable fabrication of complex microarchitectures, such as vascular-like channels, but material options are limited and photoinitiator cytotoxicity must be considered (Quan et al., 2020). Cure depth and feature fidelity are influenced by energy dose and initiator/inhibitor kinetics, with staged post-curing enhancing modulus at the cost of brittleness. Selective laser sintering (SLS) processes polymer, ceramic, and composite powders without support structures, making it attractive for load-bearing repair (Awad et al., 2020). For example, hydroxyapatite/polymer composites fabricated by SLS show improved compressive strength and osseointegration (Bauer et al., 2016). Moreover, the fine microfeatures achievable by SLA and DLP support endothelial cell alignment and vessel sprouting, thereby enhancing angiogenic coupling and integration with surrounding tissue (Alparslan and Bayraktar, 2025; Tao et al., 2022).

### 2.3 Low-temperature deposition manufacturing

LDM extrudes inks onto a chilled platform and then employs vacuum freeze-drying or solvent sublimation to generate highly porous constructs (Liu et al., 2017; Sun et al., 2023). Because printing and phase separation occur concurrently, LDM yields hierarchical porosity from micro- to nanoscale that supports cell adhesion and tissue ingrowth while preserving intrinsic bioactivity by avoiding thermal load (Xiong et al., 2002). The method also facilitates multi-material builds for bone-defect repair by co-optimizing composition and architecture (Zafeiris et al., 2021). Inadequate solvent exchange or non-ideal freezing trajectories can lead to residual solvent, shrinkage, or pore collapse; these issues are mitigated by optimizing solvent ratios and cooling rates and by using secondary crosslinking or post-infiltration to restore mechanics without sacrificing transport pathways (Figure 3e) (Tan et al., 2023). The resulting hierarchical pores provide multiscale channels that facilitate nutrient diffusion, ion exchange, and vascular ingrowth, collectively promoting osteogenesis and accelerated defect bridging (Sun et al., 2023; Wang et al., 2021).

An additional trajectory of technological evolution is 4D printing, where stimuli-responsive materials impart time-dependent functionality to printed constructs. Incorporating such dynamic behaviors into scaffold design offers the potential to better mimic bone remodeling and adapt scaffold mechanics during different healing stages, and thus may inform future choices of printable chemistries and multi-phase architectures (Gladman et al., 2016; Lai et al., 2024; Rahimnejad et al., 2024).

### 2.4 Bioprinting

Bioprinting deposits cell-laden hydrogel inks—via extrusion or laser-assisted methods—to create living constructs that deliver cells, growth factors, and functional ions (Maresca et al., 2023; Zhou X. et al., 2021). Extrusion-based platforms (micro-extrusion/DIW) accommodate hydrogel inks and polymer/ceramic pastes but impose shear stresses that may reduce viability, motivating careful matching of ink rheology and print speed to cellular tolerance (Figure 3f) (Zhang et al., 2021). While DIW-style routes are cost-effective and high-throughput for low-viscosity inks, they often suffer from limited feature fidelity and weak initial mechanics; staged or orthogonal crosslinking can mitigate these deficits (Verma et al., 2023). Laser-assisted bioprinting offers high precision and single-cell patterning but at higher cost and lower throughput, constraining broader adoption unless micron-scale accuracy is essential (Zandrini et al., 2023).

Importantly, recent advances push bioprinting beyond static cell-laden constructs toward more physiologically faithful biofabrication. Contemporary bio-inks increasingly incorporate living cells, tailored extracellular matrices, and controlled-release factors to create spatially organized microenvironments that concurrently support osteogenesis and angiogenesis. These developments emphasize the merging of materials engineering with cellular biology and motivate design choices that account for cell viability, rheology-compatible printability, and staged crosslinking strategies (Chen Z. et al., 2023; Heid and Boccaccini, 2020; Li et al., 2020). In particular, cell-laden bio-inks enable the co-delivery of osteogenic and angiogenic cues while mitigating early inflammatory responses, providing a biologically active platform for coordinated bone regeneration (Freeman et al., 2022; Yang et al., 2022).

Overall, the main FDM techniques each demonstrate unique advantages and limitations, reflecting trade-offs between resolution, mechanical properties, material versatility, and biological function. Their success in bone repair ultimately depends on whether the chosen process can be matched with suitable biomaterials to achieve both printability and long-term regenerative performance. These fabrication biology correlations further clarify how process parameters determine microstructural outcomes that, in turn, dictate cellular behavior and functional bone regeneration.

## 3 3D printing materials

### 3.1 Bioceramics

Bioceramics are widely applied in bone tissue engineering because of their chemical similarity to the mineral phase of bone and their excellent osteoconductivity (Jiang et al., 2020). Among them, hydroxyapatite (HAp) and β-tricalcium phosphate (β-TCP) are the most representative. HAp provides high stability and long-term integration but degrades slowly, which may hinder timely replacement by new bone (Wei et al., 2022). In contrast, β-TCP resorbs more rapidly, aligning better with the time window of bone remodeling, although it exhibits lower initial mechanical strength. Combining HAp and β-TCP into biphasic calcium phosphate (BCP) achieves a balance between stability and resorption.

Bioactive glass (BG) represents another important class, releasing soluble silica species that induce a surface HAp-like layer and promote osteogenic recruitment (Baino et al., 2016; Wu et al., 2020). However, its brittleness and slow degradation restrict structural applications. Modified formulations, such as Sr-doped or CaP–BG hybrids, improve osteogenic signaling and adjust dissolution kinetics to extend processing latitude and healing windows (Demir-Oguz et al., 2023; Silva et al., 2023).

Other ceramics such as alumina (Al_2_O_3_) and zirconia (ZrO_2_) exhibit excellent hardness, wear resistance, and long-term biocompatibility, making them useful in joint and dental applications. Yet their limited bioactivity and lack of controlled degradability confine their role in scaffolds requiring both load-bearing and regeneration (Kamboj et al., 2023; Punj et al., 2021).

Calcium phosphates (CaPs) are attractive for their osteoconductivity and resorbability. 3D printing techniques allow fabrication of CaP-based scaffolds, and combining them with polymers (PLA, PLGA, PCL) or nanofillers such as Mg and graphene enhances mechanics while tuning degradation toward an 8–12 weeks window (Golzar et al., 2020; Li et al., 2024; Zarei et al., 2024). β-TCP is more readily resorbed and clinically favored over α-TCP, and both benefit from pore sizes in the hundred-micrometer range to balance transport and stability (Han et al., 2024; Jasser et al., 2021; Wang et al., 2024). Clinically, phase fraction and pore geometry should be adjusted to remodeling rates, with monetite and related phases considered when faster substitution is required (Garimella et al., 2024; Wang et al., 2024; Zhou H. et al., 2021).

Bioceramics such as HAp, β-TCP/BCP, BG, and CaPs provide excellent osteoconductivity and chemical resemblance to bone minerals, while Al_2_O_3_ and ZrO_2_ ensure durability in specific contexts. However, their intrinsic brittleness and limited control over degradation constrain their independent use in complex defects. These limitations have directed increasing attention toward polymer–ceramic hybrids and, more recently, to bioactive metals and ions that can complement ceramics by offering tunable mechanical and biological functions.

Recent studies have extended ceramic incorporation from micro- to nano-scale, significantly enhancing interfacial bonding, mechanical reinforcement, and biological signaling (Piatti et al., 2024). Nano-structured ceramics and polymer–ceramic nanocomposites provide osteoinductive topographies and modulate local ion exchange at the scaffold–tissue interface, promoting osteogenesis and angiogenesis (Ellermann et al., 2023). In parallel, gradient and multi-material ceramic scaffolds enable spatial control of composition, stiffness, and degradation, better matching the hierarchical and site-specific demands of native bone (Peng et al., 2023). Furthermore, ion-substituted bioactive glasses (e.g., Li^+^, Cu^2+^, Co^2+^) impart pro-osteogenic, pro-angiogenic, and antibacterial properties, broadening the therapeutic scope of polymer–ceramic composites for bone regeneration (Zhang et al., 2023). Collectively, these strategies represent cutting-edge directions toward multifunctional and clinically oriented scaffold design.

### 3.2 Biometal

Biometals and their released ions play pivotal roles in bone regeneration by regulating osteogenesis, osteoclast activity, angiogenesis, and immune responses (Fan et al., 2024). They are commonly incorporated into 3D-printed scaffolds through alloying, surface modification, or ion-doped composites, providing tunable mechanical properties and bioactivity.

Phosphate ions are indispensable for mineralization and HAp formation, participating in cellular energy metabolism while directly contributing to the inorganic phase of bone (Wang J. et al., 2024; Yu et al., 2023). Magnesium enhances osteoblast proliferation, suppresses osteoclast differentiation, and stimulates angiogenesis; it is introduced via degradable Mg alloys or incorporated into CaP cements and hydrogel systems for sustained release (Wang et al., 2020; Zhou H. et al., 2021). Manganese functions as a cofactor for antioxidant enzymes and modulates immune signaling, while Mn-doped CaP or BG scaffolds exhibit improved osteogenesis and vascularization *in vivo* (Li et al., 2024; Shan et al., 2025; Ye et al., 2023; Zhang et al., 2022). Zinc simultaneously promotes osteoblast activity, inhibits osteoclastogenesis, and provides antibacterial and antioxidative benefits; Zn alloys or Zn-doped ceramics/polymers are frequently employed for bone-regenerative scaffolds (Huang et al., 2024; Xu et al., 2024; Zhao et al., 2024).

Silicate ions derived from bioactive glasses or silicate-incorporated hydrogels stimulate collagen synthesis, matrix mineralization, and angiogenesis, supporting both early and long-term bone healing (Jugdaohsingh et al., 2008; Liu et al., 2025; Nielsen, 2009). Strontium exerts dual regulation by enhancing osteogenesis and suppressing bone resorption, while also polarizing macrophages toward a pro-healing phenotype; Sr-doped CaPs, BGs, and polymer coatings are widely explored to synchronize mechanical support with immunomodulation (Guo et al., 2020; Huang et al., 2020; Huang et al., 2024; Miao et al., 2023; Zhou et al., 2023).

Overall, biometals and their ionic species complement ceramics and polymers by offering dynamic regulation of bone remodeling and vascularization, making them integral components of multifunctional 3D-printed scaffolds.

### 3.3 Natural polymers

Natural polymers are widely applied in bone regeneration because of their excellent biocompatibility, biodegradability, and abundance (Asadi et al., 2020). They provide a favorable matrix for osteogenic adhesion, proliferation, and differentiation, yet their low mechanical strength restricts independent use in load-bearing sites (Islam et al., 2020; Yuan X. et al., 2024). Crosslinking and composite approaches are therefore commonly employed to enhance stiffness and to match degradation with tissue remodeling (Reddy et al., 2021).

Chitosan, derived from crustacean shells, supports adhesion, proliferation, and cartilage–bone repair, while also providing antibacterial properties (Hu et al., 2024; Ma et al., 2024; Wen et al., 2021). Its poor mechanics are often addressed by crosslinking or blending with gelatin and collagen (He W. et al., 2024; Li et al., 2025; Wang et al., 2025).

Collagen, the major extracellular matrix (ECM) protein in bone, provides abundant binding sites for integrins and releases non-toxic degradation products that facilitate osteoblast adhesion and proliferation (Kikuchi et al., 2004; Sorushanova et al., 2019). In addition, its fibrillar architecture allows incorporation of bioactive molecules such as growth factors, which further promote osteogenic differentiation and matrix deposition (Lee et al., 2021). Printed collagen lattices are usually reinforced with CaP or synthetic polymers to improve mechanical strength while retaining permeability (Zhu et al., 2024).

Gelatin, a collagen derivative, retains many of these cell-recognition motifs and thus supports cell adhesion and spreading (Kuttappan et al., 2016; Ma et al., 2024). It can be processed into hydrogels with tunable degradation rates, making it attractive for controlled drug or ion delivery (Li et al., 2024). However, due to its inherently weak mechanical properties, gelatin-based scaffolds generally require additional crosslinking or compositing with ceramics or synthetic polymers to maintain structural stability during *in vivo* implantation (Chuang et al., 2024; Li et al., 2024).

Hyaluronic acid (HA), naturally present in cartilage and synovial fluid, enhances hydration, reduces inflammation, and promotes mesenchymal stem cell migration, yet needs reinforcement due to weak mechanics (Cui et al., 2021; Hwang et al., 2023; Zhang et al., 2024; Zheng et al., 2023).

Cellulose, after chemical modification, exhibits improved rigidity and controlled degradability (Abourehab et al., 2022; Tang et al., 2024). It has been employed as a reinforcing component in hydrogel or polymer matrices, where its high aspect-ratio fibrils contribute to mechanical stability (Cui et al., 2023; Yang J. et al., 2024). Nevertheless, its intrinsic osteoinductive capacity is limited, so cellulose is often combined with bioactive ions or growth factors to enhance biological performance (Cui et al., 2023; Yang S. Y. et al., 2024).

Alginate, from brown algae, forms hydrogels upon Ca^2+^ crosslinking and serves as a carrier for cells and factors, though its low strength necessitates blending with ceramics or polymers (Abasalizadeh et al., 2020; Ahmad et al., 2021; Sarker et al., 2018; Yu et al., 2023).

**FIGURE 4:**
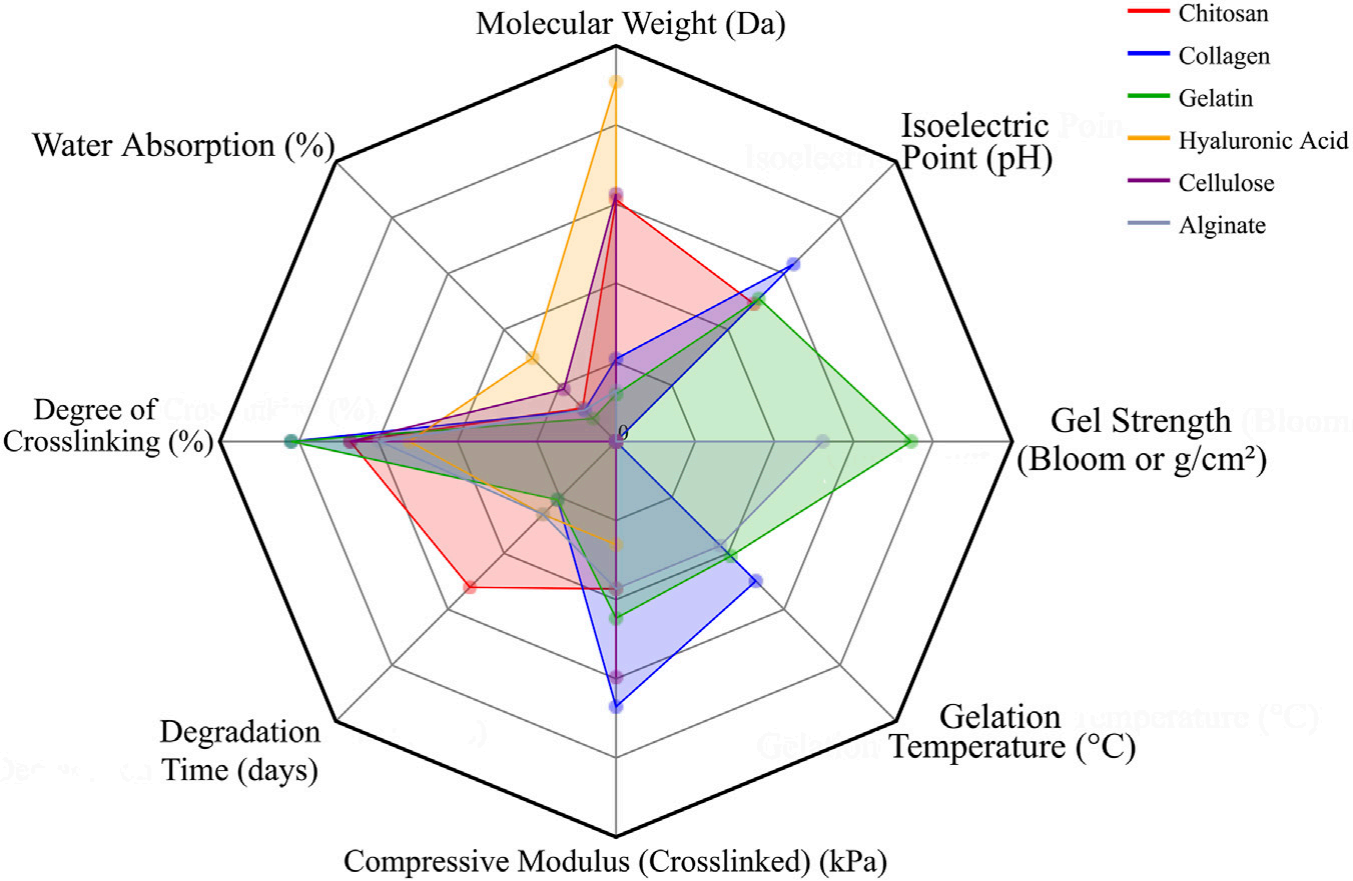
Radar chart depicting the fundamental properties of representative natural polymers used in bone tissue engineering. The relative scales of different parameters were normalized to ensure visual coherence in the chart.

Overall, natural polymers such as chitosan, collagen, gelatin, HA, cellulose, and alginate play crucial roles in creating bioactive, cell-supportive matrices, but their poor mechanics and variable degradation confine their standalone use. Integration with synthetic polymers or ceramics remains essential to provide both structural integrity and biological functionality in 3D-printed scaffolds. Notably, differences in molecular weight, degradation profiles, and mechanical performance among these polymers further emphasize the importance of material selection and design optimization (Table 1; Figure 4).

**TABLE 1:** Comparison of typical natural polymers.

Performance	Chitosan	Collagen	Gelatin	Hyaluronic acid	Cellulose	Alginate
Polymer						
MW (Da)	5.0E+04 ∼2.0E+06	3.0E+05 ∼4.0E+05	1.0E+05 ∼3.0E+05	5.0E+04 ∼3.0E+06	1.0E+05 ∼2.0E+06	3.2E+04 ∼4.0E+05
IP (pH)	6.2–7.0	7.6–9.3	4.7–9.0	—	—	—
GS (Bloom or g/cm^2^)	No Standard Bloom Value (weak gel)	No Standard Bloom Value (weak gel)	200–300	No Standard Bloom Value (Cross-linking Required)	No Standard Bloom Value (Cross-linking Required)	50–300 (Ionic Crosslinking)
GT (°C)	No Definite Gel Points	30–37	25–30	No fixed gel points	No Natural Gel Points	25 (Room Temperature)
CM (kPa)	100–400	100–800	100–500	50–300	200–600	100–400
DT (days)	14–56	7–21	7–21	7–28	90–365+	7–28
DC (%)	20–70	30–80	30–80	10–60	20–70	20–60
WA (%)	400–1,200	500–1,000	300–800	1,000–3,000	500–2000	500–1,000
Ref	Dash et al. (2011), Rinaudo (2006)	Ferreira et al. (2012), Gelse et al. (2003)	Yousefi-Mashouf et al. (2022)	Jeon et al. (2007)	Klemm et al. (2011)	Lee and Mooney (2012)

MW, molecular weight; IP, isoelectric point; GS, gel strength; GT, gelation temperature; CM, compressive modulus; DT, degradation time; DC, degree of crosslinking; WA, water absorption.

### 3.4 Synthetic polymers

Synthetic polymers are widely applied in bone regeneration because of their controllable degradation rates, favorable mechanical properties, and excellent processability. They provide stable structural support for bone defect repair but lack the intrinsic bioactivity of natural polymers, necessitating surface modification, incorporation of bioactive factors, or compositing with inorganic phases to enhance osteoinductive potential.

Poly (lactic-co-glycolic acid) (PLGA) exhibits good biocompatibility and undergoes hydrolytic degradation into lactic and glycolic acids that are cleared through endogenous metabolism. Local acidity may accumulate, so calcium phosphate (CaP) is often incorporated into buffer pH and sustain osteogenesis (Cheng et al., 2024). Although PLGA lacks inherent osteoinductive activity, it is an effective carrier for drugs or growth factors (He P. et al., 2024). With moderate mechanical strength, it is frequently combined with ceramics or bioactive glasses to improve structural performance (Annaji et al., 2024; Ke et al., 2024).

Polycaprolactone (PCL) demonstrates excellent biocompatibility without provoking inflammatory responses but shows low intrinsic osteogenic activity, which can be improved by surface modifications or ceramic incorporation (Daliri et al., 2024). PCL has favorable flexibility and printability (Afza et al., 2025), with a slow degradation rate (∼2–4 years) that allows long-term support and sustained release of loaded factors or cells (Hashemi et al., 2024; Li C. et al., 2023).

Polylactic acid (PLA) degrades *in vivo* into non-toxic byproducts over periods ranging from 6 months to several years (Stepankova et al., 2024). It provides high stiffness but is relatively brittle, limiting load-bearing applications. Compositing with calcium phosphate or hydroxyapatite improves its osteoinductive potential (Sakarya et al., 2024). PLA is widely used in fixation devices and printed scaffolds, with copolymerization approaches further balancing stiffness and osteogenic performance (Guo et al., 2024; Rahatuzzaman et al., 2024).

Polyvinyl alcohol (PVA) is biocompatible but degrades slowly unless chemically crosslinked or enzymatically treated (Rivera-Hernández et al., 2021). It is commonly used as a hydrogel matrix or delivery vehicle (Chahal et al., 2016). In printed constructs, PVA is often combined with ceramics or polymers to enhance toughness and maintain pore stability during culture (Cui et al., 2020).

Polyglycolic acid (PGA) is biocompatible and degrades rapidly into glycolic acid, later metabolized to carbon dioxide and water (Feng et al., 2023). Although it offers high initial rigidity, rapid degradation and brittleness limit its long-term use. Reinforcement with fillers or layered structures can improve stability while preserving transport pathways (Li et al., 2022).

**FIGURE 5:**
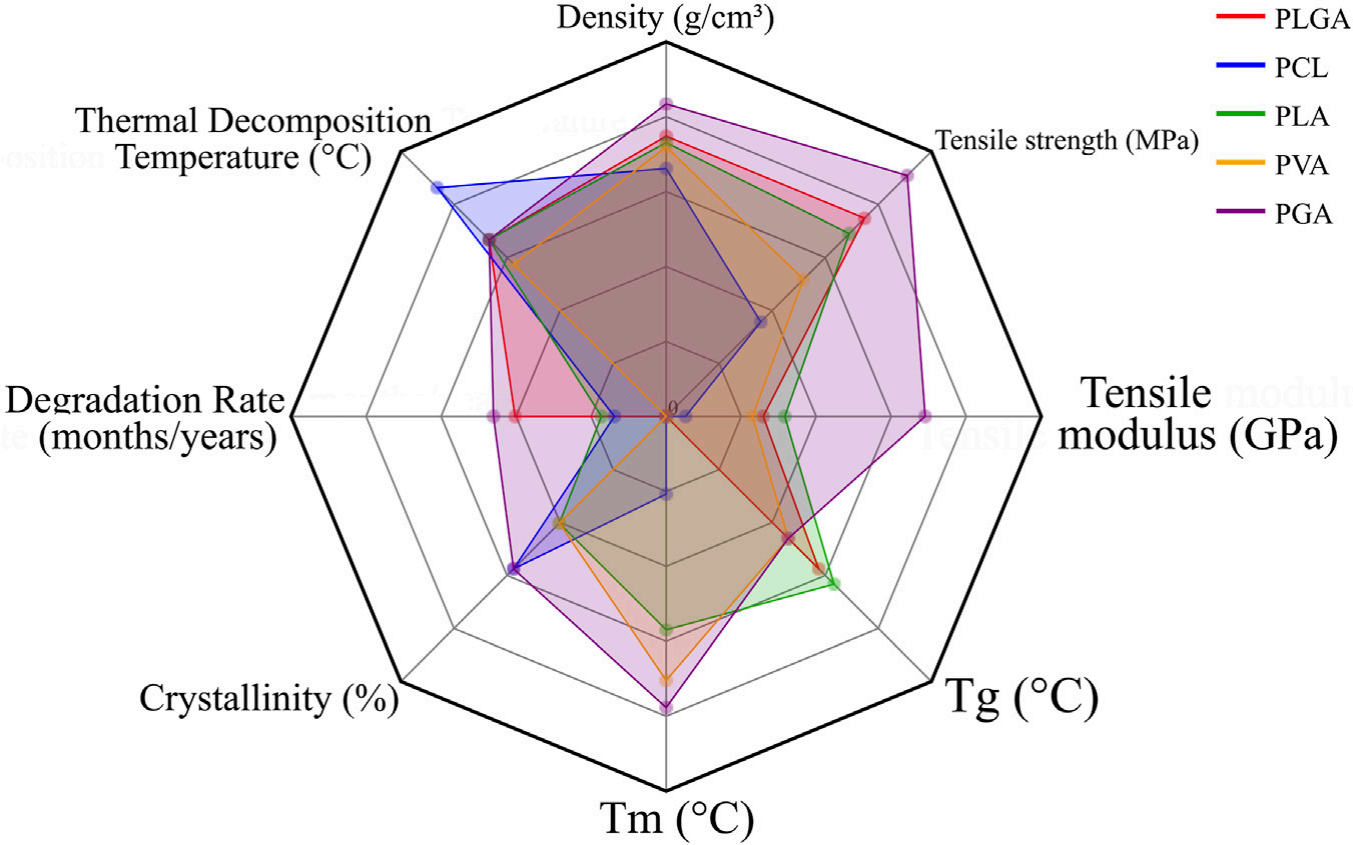
Radar chart illustrating the key properties of representative synthetic polymers applied in bone tissue engineering. To maintain consistency, parameter values were proportionally normalized across different indices for coherent visualization.

Overall, PLGA, PCL, PLA, PVA, and PGA complement each other in balancing degradation tempo, mechanical retention, and printability for bone repair. Their molecular weights, degradation profiles, and mechanical characteristics highlight the importance of coordinating material selection with processing parameters to optimize scaffold performance (Table 2; Figure 5).

**TABLE 2:** Comparison of typical synthetic polymers.

Polymer	Density (g/cm^3^)	TS (MPa)	TM (GPa)	Tg (°C)	Tm (°C)	Crystallinity (%)	DR (months/years)	TDT (°C)	Ref
PLGA	1.25–1.35	50–80	1.5–3.0	40–60	—	Amorphous	1–6 months	280–300	Wubneh et al. (2018)
PCL	1.1–1.2	20–42	0.3–0.6	−60∼–54	58–63	40–60	>24 months	350–400	Farah et al. (2016)
PLA	1.24–1.30	50–70	2.0–3.5	50–60	150–180	30–40	6–24 months	280–300	Farah et al. (2016)
PVA	1.19–1.31	30–60	1.5–2.5	30–50	180–228	30–40	Slow	230–270	Acar et al. (2014)
PGA	1.4–1.5	60–98	5.0–7.0	35–45	220–230	45–55	2–6 months	280–300	Farah et al. (2016)

TS, tensile strength; TM, tensile modulus; *Tg*, glass transition temperature; *Tm*, melting temperature; DR, degradation rate; TDT, thermal degradation temperature.

In addition to the polymeric, ceramic, and composite systems discussed above, recent studies have extended 3D printing to high-performance structural materials such as polyetheretherketone (PEEK) and titanium (Ti) lattices. Through surface engineering and hierarchical design, these materials offer exceptional mechanical reliability while exhibiting tunable biological potential, thereby bridging load-bearing stability with biofunctional integration.

## 4 Key functional attributes of 3D-printed bone scaffolds

The clinical success of 3D-printed bone scaffolds depends on their ability to integrate robust mechanical support, predictable degradation, and bioactivity within complex tissue environments. Beyond basic load bearing, scaffolds are now expected to actively coordinate their physical, chemical, and biological properties to guide new bone formation, resist infection, and promote vascularization. These interrelated functions—physical strength, degradation behavior, biological performance, antimicrobial properties, and angiogenic capacity—form the basis for evaluating and optimizing next-generation constructs. This review summarizes recent progress in materials, fabrication strategies, and functional optimization of 3D-printed scaffolds, with a particular emphasis on the interplay among these core performance criteria.

### 4.1 Physical strength

Mechanical performance is essential for 3D-printed bone scaffolds: they must provide early fixation under physiological loading while preserving interconnected porosity for nutrient transport and tissue ingrowth. Target properties depend on defect location and load-sharing requirements; therefore, material selection (polymer, ceramic, composite, or advanced systems such as PEEK and Ti) and architectural design (pore size, strut thickness, lattice topology) must be carefully coordinated within the printable processing window to avoid trade-offs between structural fidelity and biological function.

**FIGURE 6:**
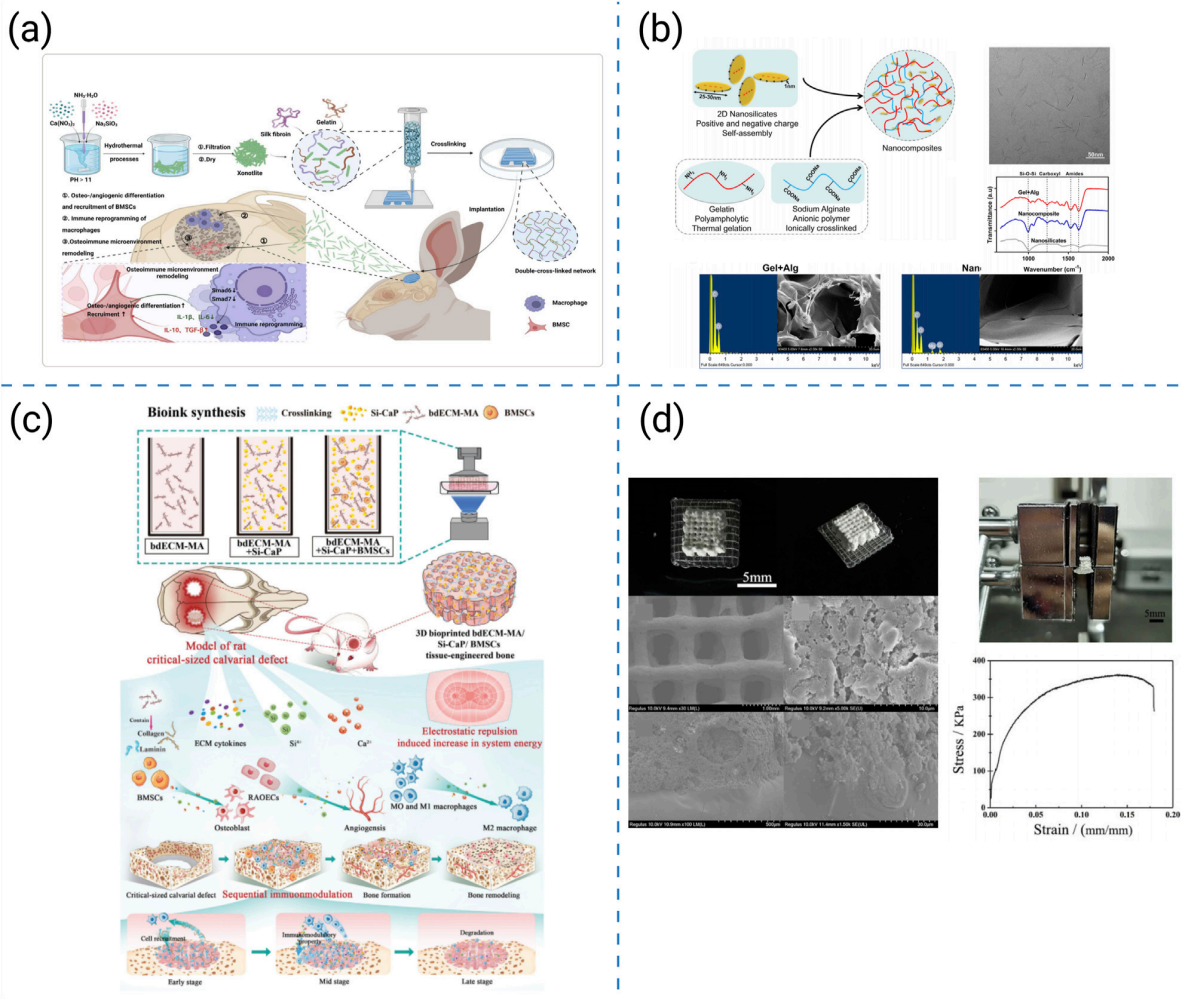
**(a)** 3D-printed hydrogel scaffolds reinforced with sepiolite nanofibers (Yang S. Y. et al., 2024). **(b)** Preparation and physical characterization of the nanocomposite hydrogels (Liu et al., 2020). **(c)** Construction and investigation schematic of the DLP 3D-bioprinted bdECM-MA/Si-CaP/BMSC tissue-engineered bone with exceptional mechanical strength (Liu et al., 2024). **(d)** Macrostructure and microstructure of the CDHA/PLGA bilayer scaffold in photographs and SEM images (Wu et al., 2021).

Current strategies to enhance the mechanical strength of 3D-printed biomaterials can be broadly classified into four categories: (i) nanofiller reinforcement, (ii) polymer-ceramic hybridization, (iii) structural topology optimization, and (iv) interfacial cross-linking, which together encompass the principal approaches reported in recent studies. Figure 6 summarizes four representative strengthening methods, each illustrating a distinct approach to enhancing the physical and biological strength of 3D-printed scaffolds.

Reinforcement strategies preserving printability have therefore been widely investigated, such as the following representative examples. Yang SY et al. introduced xonotlite nanofibers into silk-gelatin hydrogels, increasing the compressive modulus from 3.4 kPa to 57 kPa while preserving 600 μm pores and >65% porosity; these mechanics coincided with pro-osteogenic, pro-angiogenic, and osteoimmune benefits (Figure 6a) (Yang S. Y. et al., 2024). Liu B et al. enhanced the mechanical robustness of hydrogel inks by incorporating nanosilicates. This increased compressive strength and reduced creep, while maintaining lattice fidelity in cell-laden constructs, thereby accelerating calvarial defect healing *in vivo* (Figure 6b) (Liu et al., 2020). By incorporating ceramic nanoparticles into bdECM-MA, Liu et al. achieved MPa-level compressive strength without loss of extrusion fidelity and demonstrated sequential immunomodulation that promoted bone bridging (Figure 6c) (Liu et al., 2024). Wu et al. fabricated CDHA–PLGA bilayers that distributed loads, retained toughness and fatigue resistance for partial load-bearing repair, and supported *in vivo* osteogenesis in rabbit cortical-defect models (Figure 6d) (Wu et al., 2021).

These representative studies illustrate different routes toward reinforcing printable scaffolds. Comparatively, nanofiller reinforcement provides a scalable means to increase stiffness but may compromise diffusion at high filler content. Polymer–ceramic hybridization achieves a balance between strength and biological compatibility but often requires delicate interfacial design. Structural topology optimization enhances load distribution through geometric design, although its success depends on printing precision and scale fidelity. Interfacial cross-linking improves stress transfer and fatigue resistance, yet excessive crosslinking can reduce elasticity. Overall, combining these strategies can yield multi-scale reinforcement that couples mechanical robustness with biological functionality.

Beyond polymer–ceramic systems, material-specific strengthening pathways have also gained prominence in recent studies. For high-performance polymers such as PEEK, surface activation and hierarchical porosity engineering represent effective strategies to enhance interfacial load transfer and osseointegration while preserving the polymer’s intrinsic fatigue resistance and radiolucency. Notably, magnesium surface-activated 3D-printed porous PEEK scaffolds have shown markedly improved fixation and *in vivo* bone integration (Wei et al., 2023). In parallel, topology-optimized Ti lattices offer a metallic route to mechanical strengthening. By tailoring lattice architecture and porosity gradients, Ti scaffolds achieve tunable effective modulus and MPa-level load-bearing capacity while mitigating stress shielding, thus providing a biomimetic mechanical environment conducive to osseointegration (Wang et al., 2022).

Collectively, these PEEK and Ti-based pathways complement the four generic strengthening strategies, expanding the design framework for achieving a balance between mechanical reliability and biological performance in 3D-printed bone scaffolds.

### 4.2 Degradation behavior

Controlled degradation is critical for 3D-printed bone scaffolds: they must maintain structural integrity during early fixation yet gradually resorb in concert with tissue regeneration to avoid long-term residue or mismatch with remodeling rates. The tempo of degradation depends on both material chemistry and scaffold architecture, requiring careful synchronization with biological milestones.

**FIGURE 7:**
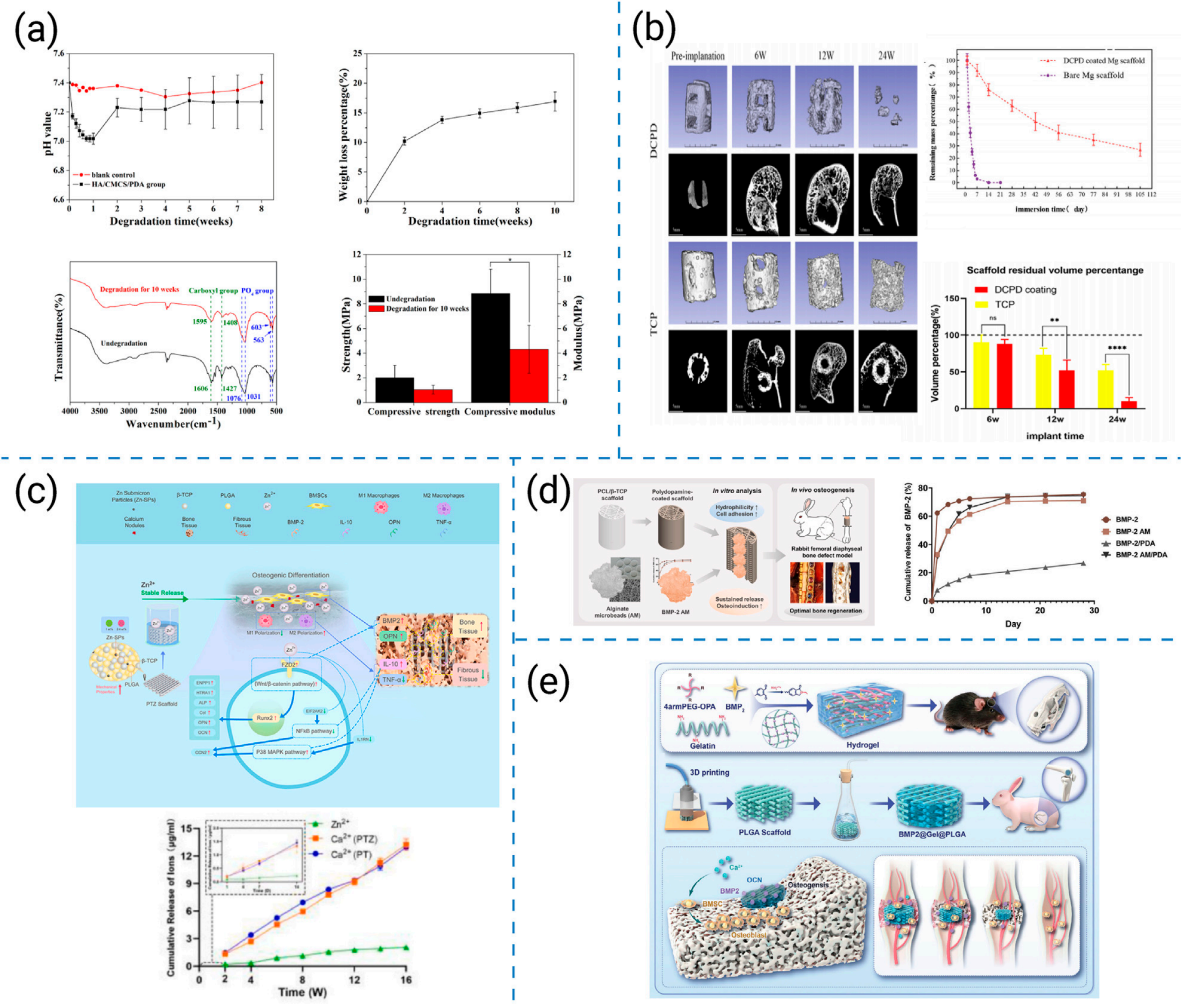
**(a)** Degradation performance of 3D-printed HA/CMCS/PDA porous bio-scaffolds (Chen et al., 2020). **(b)** Degradation performance of DCPD-coated 3D-printed porous magnesium scaffolds (Zhang et al., 2022). **(c)** Schematic illustration, potential molecular mechanisms, and cumulative release profiles of Ca^2+^ and Zn^2+^ from low-temperature 3D-printed Zn-SPs/PLGA/β-TCP scaffolds (Li X. et al., 2023). **(d)** Schematic illustration and release kinetics of 3D-printed PCL/β-TCP scaffolds coated with PDA and AM and loaded with BMP-2 (Lee et al., 2024). **(e)** 3D-printed porous PLGA composite scaffolds loaded with BMP-2/4armPEG-OPA/gelatin hydrogels (Dong et al., 2025).

Recent strategies for regulating scaffold degradation can be broadly categorized into five major approaches: (i) compositional modification through bioactive fillers or polymer blending, (ii) surface coating and interfacial cross-linking to modulate dissolution kinetics, (iii) ion incorporation for biochemical regulation, (iv) growth-factor-assisted degradation coupling, and (v) hybrid systems integrating hydrogel components for spatiotemporal control. Representative studies for each approach are summarized in Figure 7.

Based on these approaches, researchers have developed representative composite and surface-modification strategies as follows. Chen T et al. incorporated hydroxyapatite (HAp), carboxymethyl chitosan (CMCS), and polydopamine (PDA) into printed scaffolds, stabilizing degradation and creating a bioactive microenvironment that fostered osteoconduction (Figure 7a) (Chen et al., 2020). Zhang Y et al. designed porous magnesium scaffolds coated with calcium phosphate, regulating rapid Mg corrosion and aligning degradation with new bone formation; this in turn enhanced *in vivo* osteointegration (Figure 7b) (Zhang et al., 2022). Li C et al. fabricated PLGA/β-TCP scaffolds doped with Zn^2+^ ions, achieving sustained release that simultaneously promoted osteoinduction and attenuated inflammatory responses, thus extending the functional healing window (Figure 7c) (Li X. et al., 2023). Lee S et al. constructed PCL/β-TCP scaffolds with polydopamine coatings and alginate microbeads encapsulating BMP-2, facilitating sustained growth-factor delivery while improving surface wettability and cell affinity (Figure 7d) (Lee et al., 2024). Dong R et al. integrated low-polymer-content hydrogels into PLGA scaffolds, allowing local and sustained BMP-2 release that accelerated large defect repair without compromising scaffold stability (Figure 7e) (Dong et al., 2025).

These representative studies illustrate distinct pathways for synchronizing scaffold degradation with bone regeneration. Compositional modifications allow broad tunability but may cause unpredictable by-products. Surface coatings and interfacial cross-linking provide precise control of degradation rates, yet can delaminate under dynamic loading. Ion incorporation modulates chemical dissolution and cell signaling but requires strict concentration optimization. Growth-factor-assisted coupling offers spatiotemporal coordination between biochemical signaling and scaffold resorption, whereas hydrogel–polymer hybrids further enable local degradation control and biological integration. Together, these approaches highlight complementary strengths and limitations, emphasizing the need for multi-mechanistic degradation design to sustain long-term bone regeneration.

### 4.3 Biological performance

Biological performance is fundamental to the regenerative success of 3D-printed bone scaffolds. Beyond providing mechanical stability, scaffolds must actively regulate cell adhesion, differentiation, angiogenesis, and immunomodulation to guide host responses and promote bone remodeling. These biological outcomes are governed by scaffold chemistry, ion release, architecture, and incorporated signaling molecules. Recent advances further demonstrate that even high-performance materials such as PEEK and Ti can be bioactivated through interfacial engineering and hierarchical micro/nanostructuring to enhance cellular interactions and osseointegration, thereby extending biological design strategies beyond conventional polymer–ceramic systems.

**FIGURE 8:**
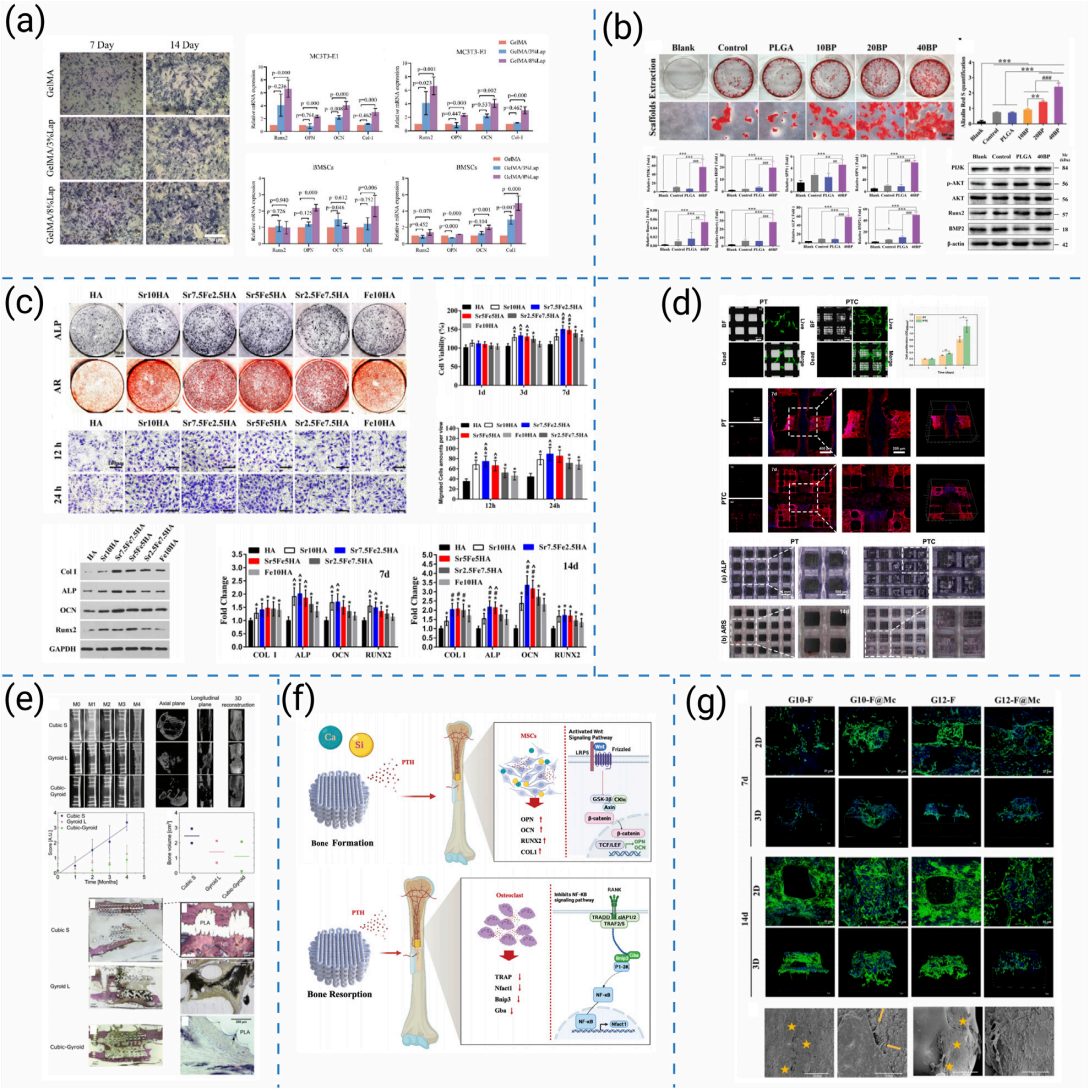
**(a)** Osteogenic differentiation assessment of 3D-printed hydrogel scaffolds (Zhou et al., 2024). **(b)** PLGA/BP scaffolds promote osteogenic differentiation and mineralization by activating PI3K-AKT signaling pathway (Long et al., 2023). **(c)** *In vitro* osteogenesis evaluations of MC3T3 cells stimulated by each group of scaffold extracts (Yang et al., 2021). **(d)** Cell viability and osteogenic differentiation on high-precision 3D-printed PCL/β-TCP hierarchical fiber scaffolds (Wang et al., 2021). **(e)** 3D-printed BMP2/PLA bio-coated scaffolds and their effects on bone regeneration (Garot et al., 2023). **(f)** Bidirectional regulation of bone homeostasis by 3D-printed PMBG/TCP/PTH scaffolds (Ren et al., 2023). **(g)** The proliferation and migration of MC3T3-E1 cells on 3D printed frameworks and composite scaffolds (Guo et al., 2023).

Current strategies to enhance the biological performance of 3D-printed scaffolds can be broadly categorized into seven representative approaches: (i) osteoimmunomodulatory regulation, (ii) microenvironmental reprogramming, (iii) bioactive-ion substitution, (iv) structural and architectural optimization, (v) macroscopic design for translational repair, (vi) biochemical cue delivery, and (vii) extracellular-matrix-mimetic functionalization. These strategies collectively target immune modulation, biochemical activation, and architectural optimization. Representative studies for each category are summarized in Figure 8.

Building on this foundation, researchers have investigated diverse strategies to enhance scaffold bioactivity. Zhou L et al. functionalized GelMA/Laponite hydrogels to recruit bone marrow-derived mesenchymal stem cells (BMSCs) through AMPK/mTOR-mediated osteoimmunomodulation, enhancing osteogenesis (Figure 8a) (Zhou et al., 2024). Similarly, Long J et al. demonstrated that PLAG/black phosphorus scaffolds could reshape the osteoimmune microenvironment and promote *in vivo* bone formation (Figure 8b) (Long et al., 2023). Beyond immune regulation, Yang L et al. introduced Sr^2+^/Fe^3+^ co-substituted hydroxyapatite into cryogenically printed scaffolds, leveraging bioactive ions to stimulate osteogenesis (Figure 8c) (Yang et al., 2021). Structural precision also contributes: Wang Q et al. produced cross-scale PCL/β-TCP scaffolds with high-fidelity fibers that supported robust cell ingrowth and intrapore bone formation (Figure 8d) (Wang et al., 2021). Translating to large-animal models, Garot C et al. designed osteoinductive polymeric scaffolds with optimized architecture that successfully repaired sheep metatarsal critical-size defects (Figure 8e) (Garot et al., 2023). Bioactivity can be further tuned through biochemical cues: Ren Y et al. integrated PTH(1–34) into photocurable PMBG/TCP scaffolds, enabling bidirectional regulation of bone homeostasis and accelerating regeneration (Figure 8f) (Ren et al., 2023). Complementarily, Guo L et al. incorporated chondroitin sulfate microspheres into printed frameworks, improving ECM mimicry and facilitating bone repair (Figure 8g) (Guo et al., 2023).

Beyond polymer-ceramic scaffolds, material-specific bioactivation strategies have been developed for high-performance polymers and metallic systems to enhance cellular interactions and tissue integration. For PEEK-based scaffolds, surface modification and micro/nanostructuring are commonly applied to overcome bioinertness, improving protein adsorption and osteogenic differentiation while preserving mechanical durability (Xu et al., 2024). For Ti lattices, bioactive coating deposition and gradient topology optimization synergistically promote osteoconduction and vascularized bone ingrowth, supporting stable osseointegration (Deering et al., 2023). Together, these polymeric and metallic bioactivation approaches expand the biological design space of 3D-printed scaffolds, integrating surface engineering with immunoregulatory and biochemical modulation frameworks.

Comparatively, osteoimmunomodulatory and microenvironment-reprogramming strategies modulate inflammatory cascades to create pro-healing niches but often involve complex cytokine dynamics. Ion substitution offers durable stimulation of osteogenesis yet requires strict dose control. Structural and architectural optimization enhances spatial guidance for tissue ingrowth but depends on printing precision. Macroscopic design facilitates clinical translation by bridging laboratory and animal-scale defects, though fabrication reproducibility remains challenging. Biochemical cue delivery provides targeted signaling yet demands stable release kinetics, while ECM-mimetic functionalization reproduces native matrix interactions but increases fabrication complexity. Collectively, these seven strategies demonstrate complementary roles, emphasizing that synergistic integration of immunoregulatory, biochemical, and structural cues is essential for achieving robust and coordinated biological performance in 3D-printed bone scaffolds.

### 4.4 Antimicrobial properties

Postoperative infection and biofilm formation are major causes of failure in bone repair, particularly in large defects requiring prolonged fixation. Beyond providing mechanical support and osteoconductivity, 3D-printed scaffolds increasingly need built-in anti-infective functions that deliver high local efficacy with minimal systemic toxicity. Effective designs combine bactericidal/anti-adhesive cues (e.g., silver or magnesium-based systems, polyphenols), controlled spatiotemporal release, and architectures that preserve porosity for nutrient transport while limiting microbial colonization without compromising osteogenesis.

**FIGURE 9:**
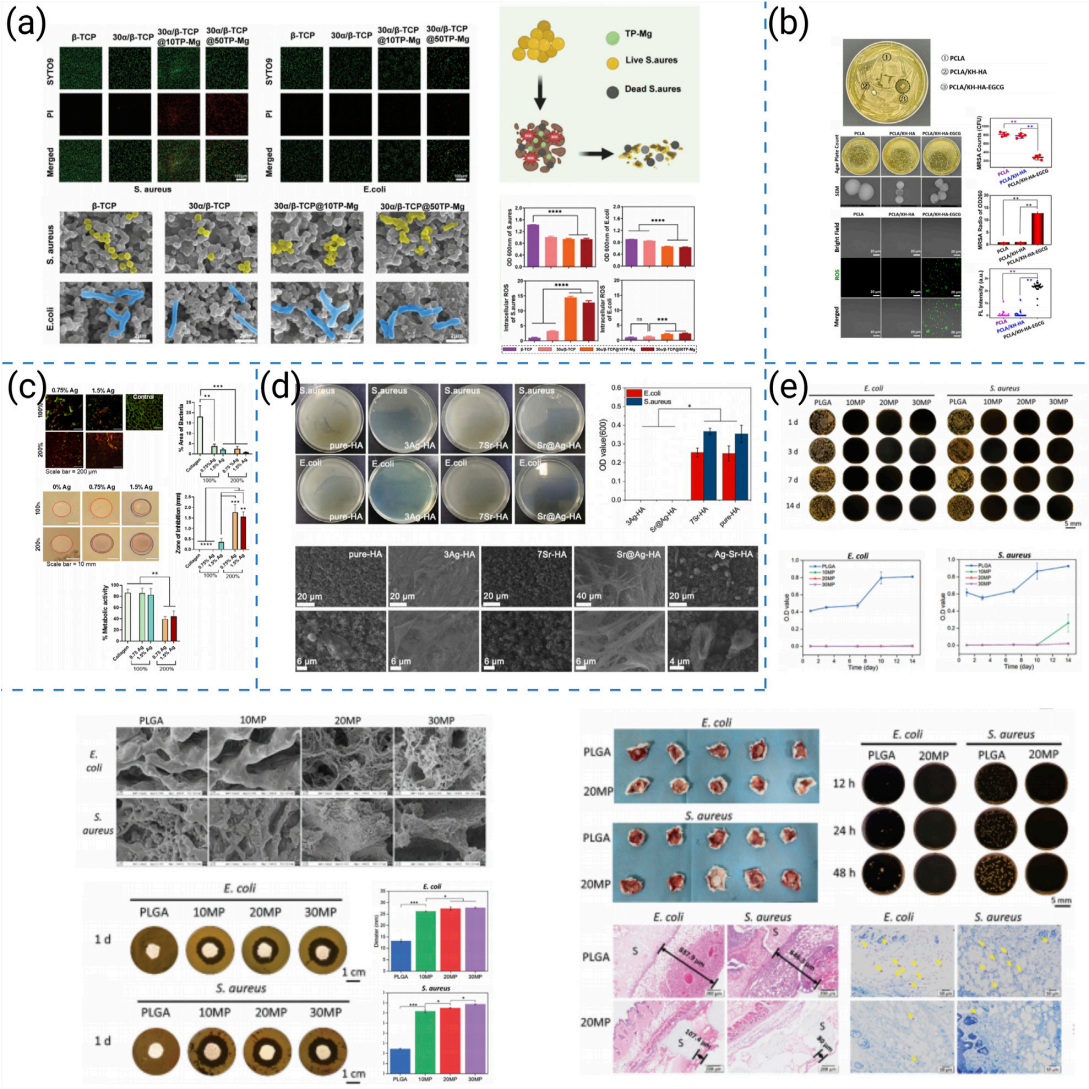
**(a)** *In vitro* antibacterial study of biomimetic α/β-TCP scaffolds loaded with TP-Mg nanoparticles fabricated by low-temperature 3D printing (Hu et al., 2024). **(b)** Antimicrobial activity of the coated scaffolds (Zhang et al., 2022). **(c)** Antibacterial properties of collagen-AgHA scaffolds *in vitro* (Genoud et al., 2025). **(d)** *In vitro* anti-bacterial effect of different scaffolds (Wang et al., 2025). **(e)** *In vitro* and *in vivo* antibacterial properties of low-temperature 3D-printed MgO_2_/PLGA porous nanoscaffolds (Li et al., 2024).

Recent anti-infective strategies for 3D-printed bone scaffolds can be broadly categorized into five major approaches:(i) locally deployable antibacterial nanoparticles, (ii) bioactive surface coatings (e.g., polyphenols or bactericidal ions), (iii) load-bearing composite frameworks incorporating Ag-doped ceramics, (iv) spatiotemporally programmed dual-release systems, and (v) time-sequenced oxygen-releasing platforms with potential adjunct antitumor activity. Representative studies for each category are summarized in Figure 9.

Building on this rationale, researchers have developed complementary anti-infective strategies within printable architectures. Hu et al. integrated TP–Mg nanoparticles into biomimetic scaffolds, achieving potent antibacterial activity and, concomitantly, enhanced osteogenesis in infectious bone-defect models (Figure 9a) (Hu et al., 2024). Zhang et al. produced PCLA scaffolds with nHA coatings doped with EGCG; the constructs supported bone growth and simultaneously inhibited colonization by multidrug-resistant bacteria (Figure 9b) (Zhang et al., 2022). Genoud et al. reinforced collagen/Ag-HA with 3D-printed frameworks to yield load-bearing constructs that prevented infection and improved repair outcomes *in vivo* (Figure 9c) (Genoud et al., 2025). Using coaxial printing, Wang et al. fabricated Sr@Ag scaffolds that delivered stepwise antimicrobial and osteogenic cues, showing efficacy against chronic osteomyelitis while supporting bone regeneration (Figure 9d) (Wang et al., 2025). In addition to direct antimicrobial action, Li et al. designed time-sequential MgO_2_/PLGA scaffolds that supported bone healing and suppressed postsurgical osteosarcoma, illustrating how anti-infective designs can be coupled with adjunct antitumor therapy (Figure 9e) (Li et al., 2024).

Comparatively, locally deployable antibacterial nanoparticles deliver high local potency with minimal systemic exposure, but dispersion stability and long-term persistence require control. Bioactive surface coatings provide contact-killing and anti-adhesive effects, yet durability and potential delamination under dynamic loading remain concerns. Ag-doped ceramic composite frameworks enable load-bearing infection control, but silver dosage must be balanced to avoid cytotoxicity while maintaining osteoconductivity. Spatiotemporally programmed dual-release systems synchronize early antibacterial action with later osteogenesis, though manufacturing complexity and release-profile calibration are non-trivial. Time-sequenced oxygen-releasing platforms expand from anti-infection to adjunct antitumor potential, but reactive-oxygen species management and tissue safety windows require careful optimization. Together, these approaches demonstrate complementary strengths and limitations, underscoring that integrating local bactericidal cues with controlled release and osteoconductive architectures is essential for durable infection suppression without compromising bone regeneration.

### 4.5 Angiogenic capacity

Vascularization plays a decisive role in the clinical success of 3D-printed bone scaffolds. Newly formed vessels not only provide oxygen and nutrients but also remove metabolic waste and deliver progenitor cells and signaling molecules, thereby tightly coupling angiogenesis with osteogenesis. Insufficient or delayed vascularization often results in poor tissue integration, necrosis in large defects, or long-term implant failure, underscoring the necessity of incorporating angiogenic regulation into scaffold design. Recent advances further highlight that even high-performance materials such as PEEK and Ti can be tailored through hierarchical architecture and bioactive surface modification to promote vascularization, extending angiogenic design principles beyond conventional polymer–ceramic scaffolds.

**FIGURE 10:**
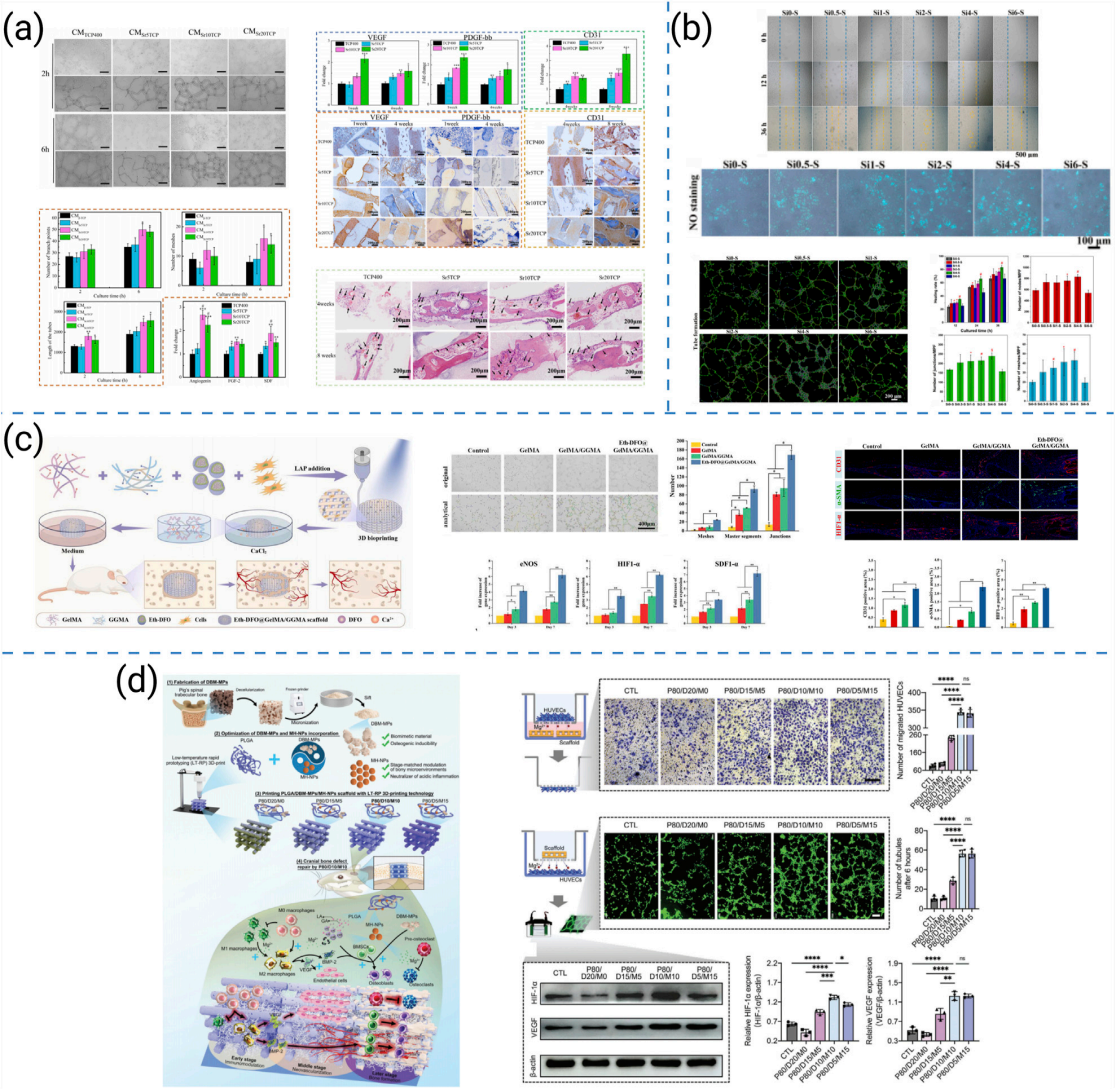
**(a)** Proangiogenic capacity of 3D-printed SrTCP highly interconnected porous scaffolds (Miao et al., 2023). **(b)** *In vitro* angiogenesis behaviors of HUVECs cultured in the extracts of Si-doped BCP scaffolds (Lu et al., 2024). **(c)** Schematic illustration for the construction process by 3D bioprinting Eth-DFO@GelMA/GGMA scaffold and its *in vitro* angiogenic capacity (Li et al., 2022). **(d)** Schematic illustration of a novel 3D-printed PLGA/DBM-MPs/MH-NPs scaffold for the enhancement of endogenous bone regeneration and *in vitro* angiogenic capacity of the four PLGA hybrid scaffolds (Yuan X. et al., 2024).

Recent pro-angiogenic strategies for 3D-printed bone scaffolds can be broadly organized into four representative approaches: (i) ion doping to stimulate pro-angiogenic signaling, (ii) hydrogel/bioink engineering with double-crosslinking to create endothelial-permissive microenvironments, (iii) stage-regulative designs that coordinate early immunomodulation, mid-stage angiogenesis, and late-stage osteogenesis, and (iv) compositional–architectural optimization to support vascular infiltration. Representative studies for each approach are summarized in Figure 10. Scaffold design must therefore integrate pro-angiogenic cues into both material chemistry and structural architecture, as illustrated by the following representative studies.

Miao et al. developed Sr-doped CaP ceramics that polarized macrophages toward pro-angiogenic phenotypes, enhanced early vascularization, and accelerated bone repair *in vivo* (Figure 10a) (Miao et al., 2023). Lu et al. optimized Si doping in BCP scaffolds and observed dose-dependent gains in osteogenic and angiogenic performance, with *in vivo* validation confirming superior neovascularization (Figure 10b) (Lu et al., 2024). Li et al. constructed gelatin/gellan-gum bioprinted scaffolds with a double-crosslinking network that improved stability and established microenvironments conducive to endothelial recruitment and vascularized bone regeneration (Figure 10c) (Li et al., 2022). Yuan et al. designed a stage-regulative scaffold that sequentially modulated early osteo-immunomodulation, mid-stage angiogenesis, and late-stage osteogenesis, integrating vascularization across the healing timeline (Figure 10d) (Yuan X. et al., 2024).

Beyond conventional ceramic and hydrogel systems, PEEK and Ti scaffolds have also been explored for promoting vascularization in load-bearing contexts. For PEEK-based scaffolds, introducing hierarchical porosity and bioactive coatings facilitates endothelial adhesion and migration while maintaining structural integrity and fatigue resistance; hierarchically porous 3D-printed PEEK scaffolds have been shown to support neovascularization and enhance osseointegration *in vivo* (Chen X. B. et al., 2023). For Ti lattices, architectural optimization combined with bioactive surface modification creates interconnected channels that promote perfusion and vessel ingrowth under physiological loading, with recent studies using additively manufactured Ti-6Al-4V lattices demonstrating mature vascular penetration and cortical–cancellous bone integration in large animal models (Feldman et al., 2024). Together, these material-specific approaches highlight that vascularization can be engineered not only through chemical cues but also through mechanical and architectural regulation in high-performance scaffold systems.

Comparatively, ion-doped ceramics provide robust pro-angiogenic cues that can accelerate early vessel formation, yet their efficacy depends on a narrow dosage window and controlled release profiles. Hydrogel/bioink systems with double-crosslinking establish endothelial-permissive microenvironments and improve structural stability, although diffusion paths and degradation rates must be carefully tuned to sustain perfusable networks. Stage-regulative scaffolds synchronize immune resolution, neovascularization, and subsequent osteogenesis, but the design and validation of temporally sequenced cues increase manufacturing complexity. Compositional–architectural co-optimization supports vascular infiltration while maintaining printability; however, achieving reproducible architectures across scales remains challenging. Taken together, these strategies present complementary advantages and limitations, indicating that integrating ionic, biochemical, and architectural cues is pivotal for achieving durable and clinically relevant angiogenesis in 3D-printed bone scaffolds.

## 5 Challenges and future perspectives

### 5.1 Balancing material innovation and biofunctionality

Although bioactive ceramics, functionalized polymers, and biodegradable metals offer promising cues for osteogenesis, angiogenesis, and immune regulation, challenges remain in achieving a balance between mechanical reliability, degradation control, and biological signaling. Future directions emphasize ion doping strategies (e.g., Sr, Mg, Zn) and advanced functionalization to optimize this balance.

### 5.2 Integrating composite and multi-functional design

While composite scaffolds can combine structural support with osteoinduction, angiogenesis, and antibacterial functions, it remains challenging to integrate these features without compromising printability or stability. Future work should refine synergistic designs that maintain multifunctionality under physiological conditions. Beyond traditional polymer-ceramic systems, emerging high-performance and load-bearing materials are being adapted for 3D printing. Their integration through hierarchical architecture and surface functionalization opens new avenues for coupling mechanical endurance with long-term biological activity in clinical bone repair.

### 5.3 Refining 3D printing strategies for clinical needs

Low-temperature deposition, multi-nozzle systems, and *in situ* bioprinting have enabled spatially controlled architectures, yet reproducibility and scalability remain hurdles. Next-generation printing must reconcile gradient and hierarchical designs with clinical feasibility, ensuring structures better replicate bone microenvironments while remaining manufacturable.

### 5.4 Synchronizing mechanical–biological coupling

A persistent limitation is the mismatch between scaffold mechanics, porosity, and degradation with dynamic tissue regeneration. Designing scaffolds that maintain early fixation but progressively transfer load to new bone represents both a challenge and a future priority to ensure durable repair and functional remodeling.

### 5.5 Ensuring clinical translation and standardization

**FIGURE 11:**
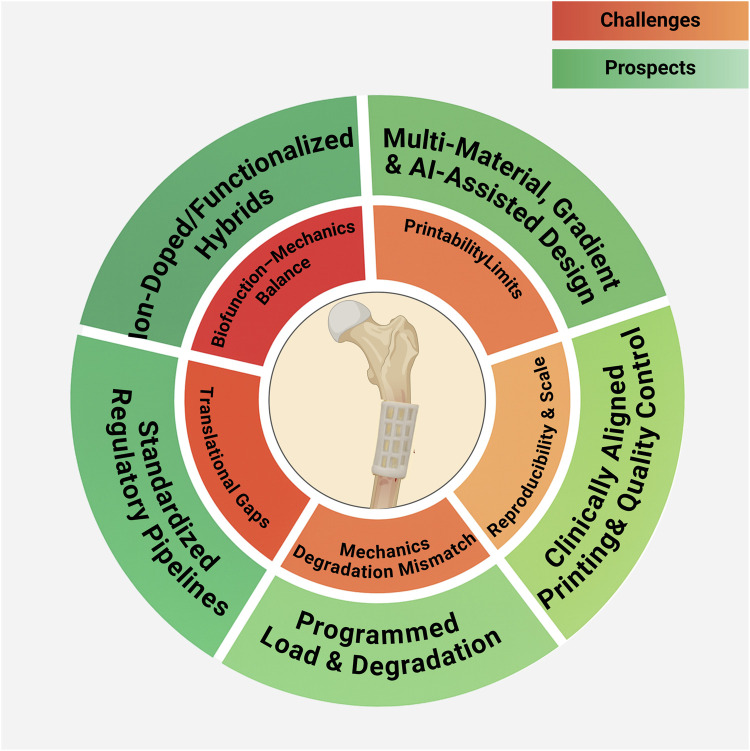
Challenges and Prospects of 3D-Printed Scaffolds for Bone Defect Repair. This figure was drawn using Biorender (https://www.biorender.com/).

Despite rapid laboratory advances, clinical translation is constrained by manufacturing reproducibility, large-scale production, and regulatory requirements. Overcoming these barriers will require standardized evaluation protocols, long-term *in vivo* studies, and coordinated efforts between academia, industry, and regulators to accelerate clinical adoption. Translational frameworks should also embrace high-performance structural systems that reconcile durability with vascularized integration, ensuring reliable performance in mechanically demanding defects. A schematic summary of the key challenges and future directions for 3D-printed scaffolds in bone defect repair is presented in Figure 11.

Finally, emerging trends highlight that AI-assisted scaffold design and deeper interdisciplinary integration are poised to accelerate clinical translation. Data-driven algorithms can optimize scaffold architecture, porosity gradients, and composite formulations to meet patient-specific mechanical and biological targets. Coupled with advances in bio-inks, dynamic (4D) materials, and multimodal imaging feedback, convergence across materials science, cellular biology, and clinical medicine will be essential to realize adaptive, personalized, and clinically translatable scaffold systems.

## 6 Conclusion

In summary, this review delineates how polymer-based, 3D-printed scaffolds can be rationally engineered by coupling material design with architectural control to achieve coordinated mechanical stability, programmed degradation, immune modulation, and angiogenic support. Across extrusion-, laser-assisted, and low-temperature routes, advances in pore geometry, interconnectivity, and anisotropy translate into improved osteoconductivity, early load transfer, and vascular ingrowth, while polymer–ceramic/ion strategies broaden bioactivity without sacrificing printability. Conceptually, this synergistic design framework clarifies how process parameters and composition determine microstructure and transport, which in turn dictate cellular behavior and functional regeneration—providing a practical map from materials innovation to translational performance.

Looking forward, priorities include 4D printing for time-dependent mechanics and remodeling, cell-inclusive bio-inks that co-deliver osteogenic/angiogenic cues under viable rheology, and AI-assisted design to optimize gradients, architectures, and composite formulations for patient-specific targets. Convergence among materials science, biology, and clinical medicine will be pivotal to convert laboratory prototypes into adaptive, personalized, and clinically translatable scaffold systems.
